# Effects of Shenfu injection on intestinal microbiota and inflammation in sepsis mice

**DOI:** 10.3389/fcimb.2025.1599903

**Published:** 2025-09-15

**Authors:** Ning Li, Fan Yi, Yujun Wang, Feng Geng, Yanli Liu, Qiushuang Liu, Yanan Guo, Ding Long

**Affiliations:** ^1^ Intensive Care Unit, The Central Hospital Wuhan, Tongji Medical College, Huazhong of University of Science and Technology, Wuhan, Hubei, China; ^2^ Key Laboratory for Molecular Diagnosis of Hubei Province, The Central Hospital of Wuhan, Tongji Medical College, Huazhong University of Science and Technology, Wuhan, Hubei, China; ^3^ Hubei Provincial Engineering Research Center of Intestinal Microecological Diagnostics, Therapeutics, and Clinical Translation, Wuhan, Hubei, China

**Keywords:** sepsis, Shenfu injection, intestinal microbiota, inflammation, traditional Chinese medicine (TCM)

## Abstract

**Introduction:**

Sepsis remains a critical challenge in intensive care medicine, necessitating novel therapeutic approaches.

**Methods:**

In this study, healthy 8-week-old male C57BL/6J mice were treated with cecal ligation and puncture (CLP) to induce a sepsis model. After successful model establishment, mice in the sham and CLP groups were injected with 200 μL of normal saline, while mice in the SFI group were injected with 200 μL of SFI. Changes in intestinal mucosal barrier function, inflammation, and intestinal microbiota were assessed in septic mice after SFI treatment.

**Results:**

SFI treatment significantly ameliorated intestinal inflammation and reduced serum levels of pro-inflammatory cytokines (IL-1β, IL-6) and renal injury markers (SCr, BUN). 16S rRNA sequencing revealed SFI-mediated gut microbial remodeling, characterized by a marked reduction in pathogenic Escherichia-Shigella abundance and concurrent enrichment of beneficial probiotics, including Akkermansia and Lactobacillus. Mechanistically, SFI exhibited dual regulatory effects on both systemic inflammation and gut microbiota homeostasis.

**Discussion:**

These findings not only validate SFI's efficacy in sepsis treatment but also propose a novel mechanism involving gut microbiome modulation. This study provides critical experimental evidence for repurposing traditional Chinese medicine in sepsis therapy and establishes a foundation for future research on microbiota-targeted interventions in critical care.

## 1 Introduction

Sepsis is a systemic inflammatory response syndrome (SIRS) caused by infection with a complex pathogenesis, and it is one of the main causes of death in critically ill patients (Meyer and Prescott, 2024; Klingensmith and Coopersmith, 2023). According to the international consensus (Sepsis-3 definition), sepsis is defined as a dysregulated host response to infection, accompanied by a Sequential Organ Failure Assessment (SOFA) score ≥2 (Seymour et al., 2016). The core pathological mechanism of sepsis is that after the invasion of pathogens, the body excessively releases inflammatory mediators and cytokines, triggering an imbalance between systemic inflammatory and anti-inflammatory responses, causing vascular endothelial damage, microcirculation disorders, and abnormal cell metabolism, ultimately leading to multiple organ failure (Dobson et al., 2024; Hotchkiss and Karl, 2003; Hunt, 2019; Shankar-Hari et al., 2016). In addition, patients with sepsis often have reduced intestinal microbiota diversity, an increased proportion of pathogenic bacteria (such as *Escherichia coli* and *Fusobacterium nucleatum*), and a decrease in beneficial bacteria (such as *Bifidobacterium* and *Lactobacillus*) (Haak and Wiersinga, 2017). Many studies have reported that the intestinal immunity plays an important role in the development of many diseases like systemic inflammatory response syndrome, and sepsis, and (multiple organ dysfunction) MODS (Brandt et al., 2022; Oami et al., 2024; Ge et al., 2020). Moreover, it was reported that Claudin-2 upregulation enhanced the intestinal permeability, immune activation, dysbiosis, and mortality in sepsis (Oami et al., 2024). Therefore, it is increasingly urgent to study the effects of sepsis on the intestinal immunity.

Intestinal microbiota is an important factor affecting intestinal immunity, imbalance of microbiota causes pathogen-associated molecular patterns (such as LPS) to enter the blood circulation through the damaged intestinal barrier, activate the Toll-like receptor (TLR4/2) signaling pathway, trigger excessive inflammatory response (such as TNF-α, IL - 6 release), and then cause systemic inflammatory response syndrome (SIRS) and MODS (Adelman et al., 2020; Kullberg et al., 2021). In addition, intestinal flora imbalance can lead to reduced expression of tight junction proteins and increased intestinal permeability, allowing bacteria and endotoxins to translocate to extraintestinal organs (Gai et al., 2021). For example, LPS released by Gram-negative bacteria enters the liver through the portal vein, activates Kupffer cells to release inflammatory factors, and aggravates sepsis-related liver damage and systemic inflammatory responses (Liu et al., 2023; Luan et al., 2024). Short-chain fatty acids (SCFAs, such as butyrate) produced by intestinal metabolism have anti-inflammatory effects, inhibit the NF-κB pathway and promote the differentiation of regulatory T cells. The level of SCFAs in the intestine of patients with sepsis is reduced, leading to imbalanced immune regulation and uncontrolled inflammatory response (Lou et al., 2023). And it was reported that the composition of the intestinal microbiome is affected by sepsis, and might contribute to the development of organ failure (Haak and Wiersinga, 2017). Therefore, restoring the balance of the microbiota is a key target for the treatment of sepsis.

Shenfu injection (SFI) is a traditional Chinese medicine containing extracts of red ginseng (Panax), aconite root (Radix aconiti lateralis preparata) and black monkshood (Aconitum) (Yang et al., 2014; Xu et al., 2024). It has been reported that SFI has a variety of anti-inflammatory, anti-apoptosis, anti-oxidation and regulation of innate immunity (Hong et al., 2015; Zhao et al., 2022). When administered clinically, it has been reported to inhibit excessive inflammatory responses (such as reducing TNF-α and IL - 6 levels) and alleviate the “inflammatory storm” in sepsis (Yang et al., 2014; Luo et al., 2021; Li et al., 2022; Xu et al., 2022). Traditionally, SFI is used to enhance myocardial contractility, dilate peripheral blood vessels, and increase blood pressure, and has the effects of improving circulation and resisting shock (Xu et al., 2024). It is reported that the mechanism of action of SFI includes restoring hemodynamic stability, increasing tissue oxygen partial pressure and oxygen content, and improving microcirculation and tissue metabolism. Therefore, SFI can promote shock resuscitation (Liu et al., 2015; Hua et al., 2024). SFI has also been shown to improve tissue function and hemodynamic status in heart failure and exert potent anti-endotoxin, anti-inflammatory effects and act as a potent oxygen free radical scavenger (Li et al., 2014; Zhao et al., 2025). In addition, studies have shown that SFI can alleviate the “inflammatory storm” in sepsis by inhibiting excessive inflammatory responses (such as reducing TNF-α and IL - 6 levels) (Jin et al., 2018; Wu et al., 2015; Xing et al., 2015; Yang et al., 2025). However, the effects of SFI on the gut microbiota remain largely unknown.

In this study, SFI was administered to septic mice to investigate its effects on gut microbiota alterations. A severe sepsis model was established via cecal ligation and puncture (CLP), followed by intravenous SFI administration. We systematically assessed intestinal epithelial integrity, inflammatory cytokine levels (e.g., IL - 1β, IL - 6) to evaluate SFI’s potential intestinal protective effects. High-throughput sequencing was employed to characterize gut microbiome dynamics, with particular attention to changes in dominant taxa. Our multimodal approach combining histopathological analysis, cytokine profiling, and 16S rDNA sequencing collectively contributes to a deeper understanding of SFI’s therapeutic mechanisms in sepsis management, particularly its role in microbiota-host crosstalk. This study provides valuable insights for developing microbiota-targeted adjuvant therapies for septic patients.

## 2 Materials and methods

### 2.1 Animals

All experiments were conducted with the consent of the hospital’s animal ethics committee with the approval number BSMS 2025 - 04-25A. Healthy 8-week-old male C57BL/6J mice (25.0g ± 5.0g) were housed in our hospital’s experimental animal center, with a temperature of 20°C ± 1°C, humidity of 50% to 60%, under a 12:12 light/dark cycle, and a ventilation rate of 8 to 15 times per hour. Using a randomized complete block design, mice were divided randomly into 3 groups of 15: sham operation group (Sham), severe sepsis group (CLP), and Shenfu injection group (SFI).

### 2.2 Animal model establishment and intervention

Severe sepsis model was induced by CLP, as previously described (Rittirsch et al., 2009). Briefly, on the day before CLP surgery, the mice were fasted for 12 hours. After weighing, they were anesthetized with 40mg/kg pentobarbital sodium. Under sterile conditions, a small amount of feces was extruded. Immediately after surgery, the animals were subcutaneously injected with 50 mL/kg body weight of physiological saline to counteract shock, and then returned to their cages. Fifteen minutes later, mice in the sham group and CLP group received 200 μL of normal saline, whereas SFI-treated animals were administered 200 μL SFI. The mice were treated through tail vein injections. After the procedure, the mice were kept in an environment at 22°C with unlimited access to food and water. They were observed until they recovered from anesthesia, then every 2 hours until 8 hours after surgery. Disease severity was assessed using the Mouse Clinical Assessment Score (M-CASS) method as reported previously (Mai et al., 2018). Briefly, the most severe sepsis is manifested by the coat being shaggy and erect, the posture being hunched, there being little or no movement, breathing being labored, eyelid and chest movements being mostly or completely closed, there being a strong fishy odor from the abdominal cavity, the intestine being obviously congested and edematous, and the ligated cecum being dark purple. Moderate sepsis is manifested by shaggy fur, an arched back, tense or stiff when disturbed or stimulated, and moderate dyspnea. Mild sepsis is manifested by normal behavior and normal appetite. The coat is slightly shaggy, activity is reduced, the back is arched, and behavior and movements are slowed, with mild dyspnea.

### 2.3 Specimen collection

Sixteen hours after surgery, all live mice were anesthetized with 0.3% pentobarbital sodium. The eye blood sample was collected as follows: the mice was fixed on the experimental table, the head and body were kept stable, the eyes were disinfected, the blood vessels were cut at the base of the eyeball with ophthalmic scissors, and then the blood was gently squeezed out. The collected blood sample was placed in a test tube containing heparin anticoagulant and gently shaken to prevent blood coagulation, then the abdominal cavity was opened. The whole intestine was removed and rinsed with PBS and then divided into two parts. One part was fixed in 4% paraformaldehyde for histological observation, and the other was used for subsequent biochemical analysis. The flushed contents of large intestine were collected in Eppendorf tubes for subsequent intestinal flora analysis. All operations were performed in a sterile environment.

### 2.4 Histological observation

Intestine tissues fixed in 4% paraformaldehyde were maintained in 10% neutral formalin buffer solution for 24h at room temperature, dehydrated in wax, and embedded in paraffin wax (JB-P5, Wuhan Junjie Electronics Co., Ltd, China). Sections were cut at a thickness of 4 μm with a paraffin sectioning machine (RM2016, Shanghai Leica Instrument, China) and stained with H&E. Observations were made with a microscope camera Nikon Eclipse E10 (Nikon, Japan).

### 2.5 Biochemical analysis

The blood samples were collected and centrifuged at 3,500 rpm for 10 minutes at 4°C and the serum was harvested. Serum interleukin (IL)-1β, IL - 6, serum creatinine (SCr), and blood urea nitrogen (BUN) were measured using enzyme-linked immunosorbent assay (Wuhan Beiyinlai Biotechnology Co., Ltd.), according to the manufacturer’s instructions.

### 2.6 DNA extraction, PCR amplification, and sequencing

Genomic DNA from the gut contents was extracted using the TIANamp Stool DNA Kit (Beijing Tiangen Biotechnology Co., Ltd.) according to the manufacturer’s instructions. Extracted DNA were assessed using 1% agarose gel electrophoresis and a NanoDrop 2000 spectrophotometer. Genomic DNA was used as a template to amplify the V3-V4 region of the 16S rRNA gene using the barcode universal primer 341F (5’-CCTACGGGNGGCWGCAG-3’) and 806R (5’-GACTACHVGGGTATCTAATCC-3’). The amplification products were sequenced by a commercial company using Illumina NovaSeq PE250.

### 2.7 Bioinformatics and data analysis

Raw data from the Illumina platform were filtered using FASTP (v0.18.0), and all obtained sequences were classified according to the corresponding unique barcode. Reads from each sample were spliced using FLASH (v1.2.7), and the spliced sequences were processed by Fastp software (Bokulich et al., 2013) to obtain high-quality reads. High-quality sequences were aligned with the Species Annotation Database (https://github.com/torognes/vsearch/) to detect chimeric sequences, and chimeric sequences were finally removed using the UCHIME algorithm (Edgar et al., 2011). Sequences were clustered into operational taxonomic units (OTUs) using UPARSE software (v7.0.1001) with 97% identity as a threshold (Edgar, 2013). Alpha diversity indices were calculated using QIIME (v1.9.1) (Caporaso et al., 2010). For the beta diversity index, the principal coordinate analysis (PCoA) was performed using the UniFrac web tool (Lozupone et al., 2006). To assess overall differences in microbial community structure, principal coordinate analysis (PCoA)and cluster analysis using Bray- Curtis distances were conducted, and microbial community functional profiles were inferred with PICRUSt version 1.0.0 (Langille et al., 2013). Bacterial community profile data were statistically analyzed with one-way analysis of variance (ANOVA) followed by a Tukey’s test in SPSS 19.0 (IBM Corporation, Armonk, NY, USA).

Data presented are pooled from three independent experiments. Values of the results are presented with mean ± SEM. Statistical analyses were performed with GraphPad Prism (v 10.0). Comparisons between two independent groups employed either the independent samples t-test (for homogeneous variances) or the Satterthwaite t-test (for heterogeneous variances). When comparing three or more independent groups, a one-way ANOVA was executed, with subsequent pairwise comparisons made using Tukey’s method. Repeated measures data were analyzed using repeated measures ANOVA. The differences were considered statistically significant at *P*<0.05.

## 3 Results

### 3.1 Establishment of a mice model of sepsis

During the 16-hour postoperative observation period, no mice died. Mice in the sham group exhibited typical behavior, were responsive, had shiny fur, regular bowel movements, and displayed no signs of intestinal hyperemia or edema. Mice in the CLP group displayed altered behavior, manifested as unkempt and upright fur, slow reactions, difficulty breathing, decreased appetite, loose stools, dark fur, and bloody ascites. The abdominal cavity emitted a strong fishy odor, the intestinal congestion and edema were obvious, and the ligated cecum was dark purple. Furthermore, mice in the SFI groups showed minimal differences in sham group. They exhibited typical behavior and maintained a normal appetite. However, they had formless stools, dull hair, and ascites.

### 3.2 The beneficial impact of Shenfu injection on the intestinal mucosal barrier in septic mice

**Figure 1:**
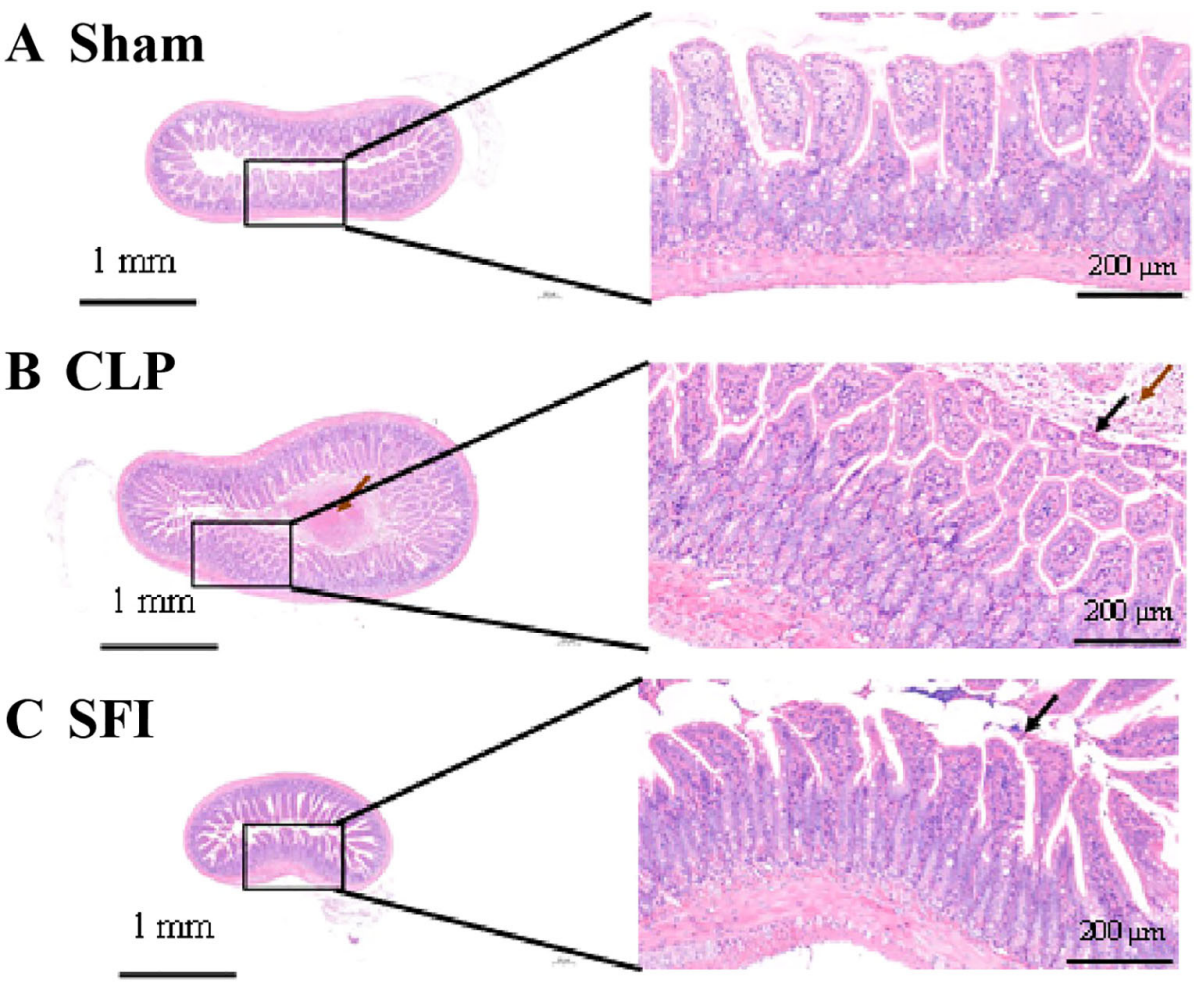
Histological comparison of intestinal tissue in three conditions: **(A)** Sham with normal tissue structure, **(B)** CLP showing tissue damage and inflammation, **(C)** SFI exhibiting reduced damage. Each condition features a small-scale image with a detailed magnified section highlighting tissue morphology. Scale bars indicate 1 millimeter and 200 micrometers.

In the sham group mice, it was observed that the intestinal villi were abundant in number and uniform in length on the surface of the intestinal tissue, with a single layer of columnar epithelium and normal morphology and structure (**Figure 1A**). However, in mice in the CLP group, mucosal epithelial cells were necrotic and sloughed off, and a large amount of necrotic cell debris was seen in the intestinal lumen (**Figure 1B**). In addition, after treatment with ginseng and aconite injection, the intestinal tissue returned to normal levels, with abundant and uniform intestinal villi on the surface, a single layer of columnar epithelium, and occasional necrosis and shedding of mucosal epithelial cells (**Figure 1C**).

### 3.3 The effects of Shenfu injection on the of mice inflammation with sepsis

**Figure 2:**
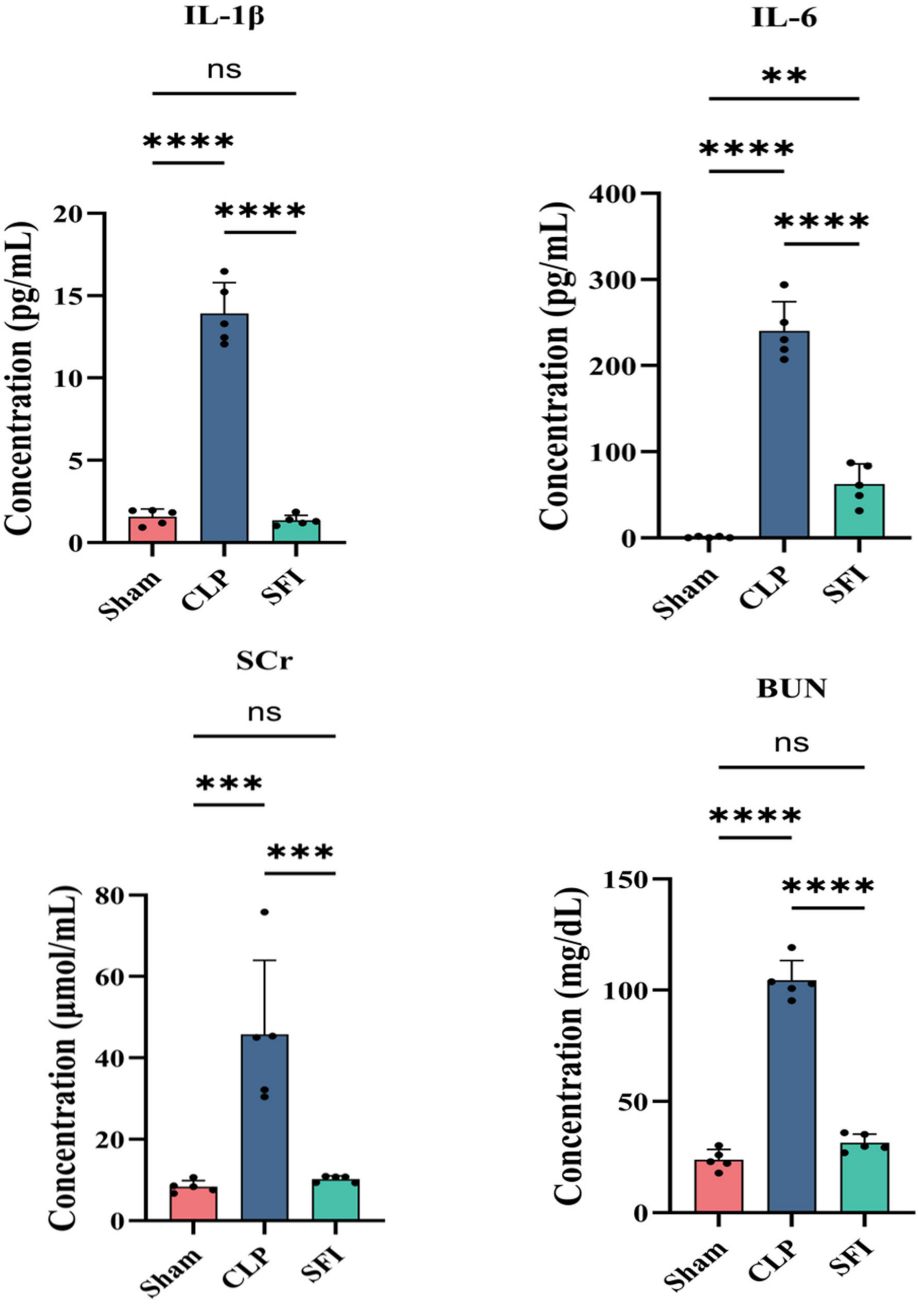
Comparison of biochemical indices in mice 16 hours after surgery. Levels of interleukin-1β **(A)**, interleukin-6 **(B)**, blood urea nitrogen **(C)** and serum creatinine **(D)** in the serum. The all data are shown as the mean±S.D. of least five independent replicates and analyzed by one-way ANOVA with Tukey’s post hoc test. P<0.01, *P*<0.001, ***P<0.0001 and and “ns” means “P>0.05”.

In order to assessment of sepsis and organ dysfunction, we studied the inflammatory factors in the blood of mice. The concentration of blood inflammatory factors after successful model establishment are shown in **Figure 2**. The results showed that compared with the sham group, the CLP group significantly increased the concentrations of IL - 1β (8.9-fold), IL - 6 (344.6-fold), SCr (5.5-fold) and BUN (4.4-fold) (*P*<0.05). However, compared with the untreated group (CLP group), shenfu administration group (SFI group) significantly reduced the concentrations of IL - 1β (10.3-fold), IL - 6 (3.8-fold), SCr (4.5-fold) and BUN (3.3-fold) (P<0.05). In addition, the concentrations of IL - 1β, IL - 6, SCr and BUN in the CLP group were significantly higher than those in the sham group (*P*<0.05), while there were no significant differences in the concentrations of IL - 1β, SCr and BUN between the SFI group and the sham group.

### 3.4 Microbial diversity

**Figure 3:**
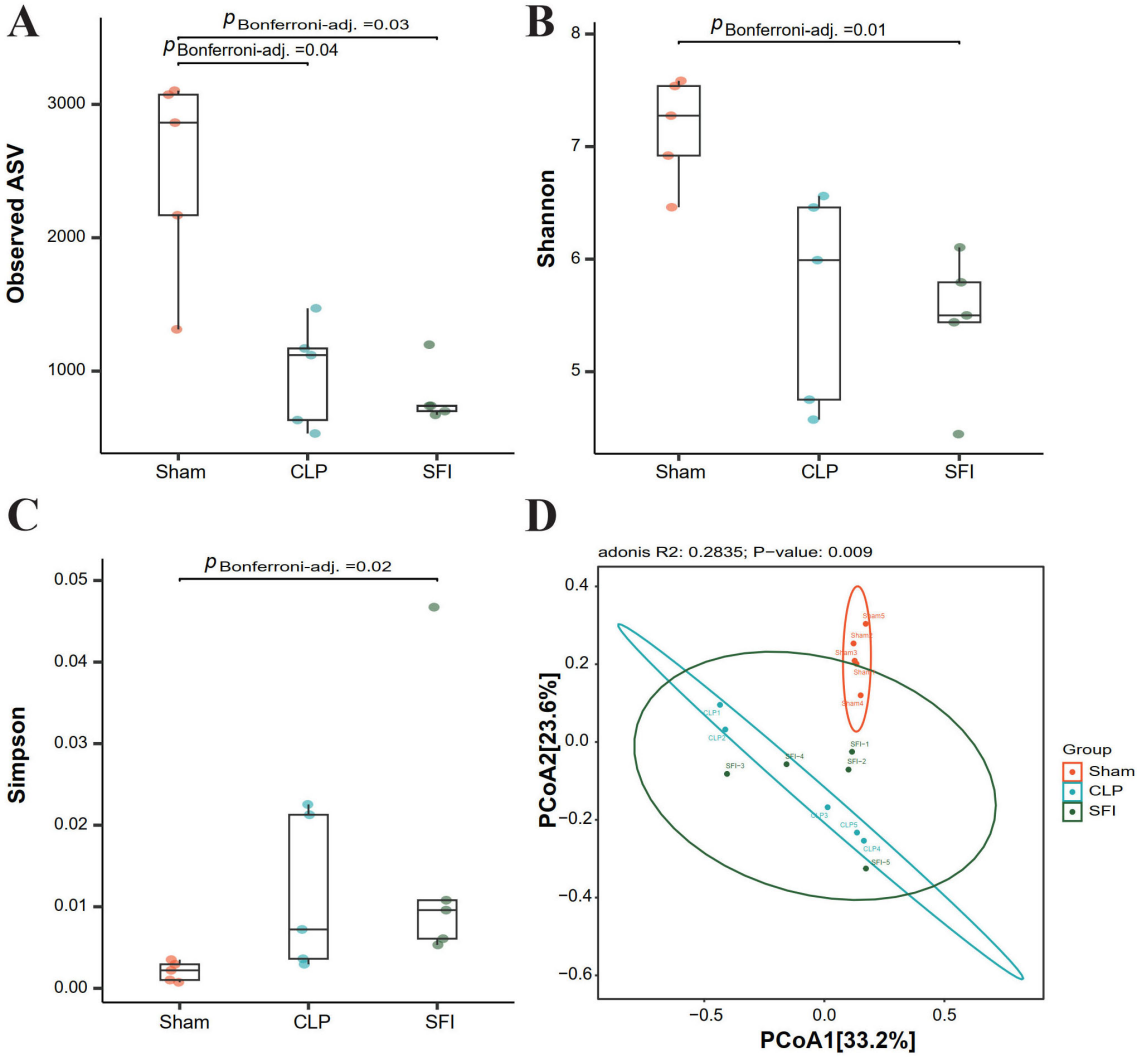
Alpha diversity estimators and bacterial community composition of gut microbiota in mice 16 hours after surgery. **(A–C)** Alpha diversity estimators: Observed ASV (A), Shannon **(B)**, and Simpson **(C)**. Differences are determined by one-way ANOVA analysis (with P < 0.05). A non-significant difference is indicated with “ns.”. **(D)** Principal coordinate analysis (PCoA) (PCoA1: 33.2% and CoA2: 23.6% of the explained variance). Each dot shows a single sample (Sham, CLP, and SFI indicate the samples from sham operation group, severe sepsis group, and Shenfu injection group, respectively).

The microbial complexity in the three groups was estimated using alpha diversity index, including richness estimators (Observed ASV) and diversity indexes (Shannon and Simpson index). The result of Observed ASV showed there were significantly differences between the sham and SFI or CLP (*P <*0.05) (**Figure 3A**), and significant differences of Shannon and Simpson Index were found between Sham and SFI (**Figures 3B, C**). Using Tukey and Wilcoxon tests, beta diversity index showed a significant difference between sham and CLP (*P* = 0.00 and 0.00, respectively), but no significant difference between SFI and sham (*P >*0.05). According to A principal coordinate analysis (PCoA) result (based on weighted UniFrac distance matrixes), the similarity among the microbial community composition of the samples were estimated (**Figure 3D**). Based on PCoA results, it was found that there were obvious differences in the bacterial community composition between the sham group compared to the CLP group, which indicated that the mice injected with CLP significantly changed the composition of intestinal microbiome, while when treated with Shenfu, more similarity was found in that composition between the sham and SFI groups, which showed that Shenfu injection could effectively regulate the disturbance of intestinal microbiome.

### 3.5 Microbial composition

**Figure 4:**
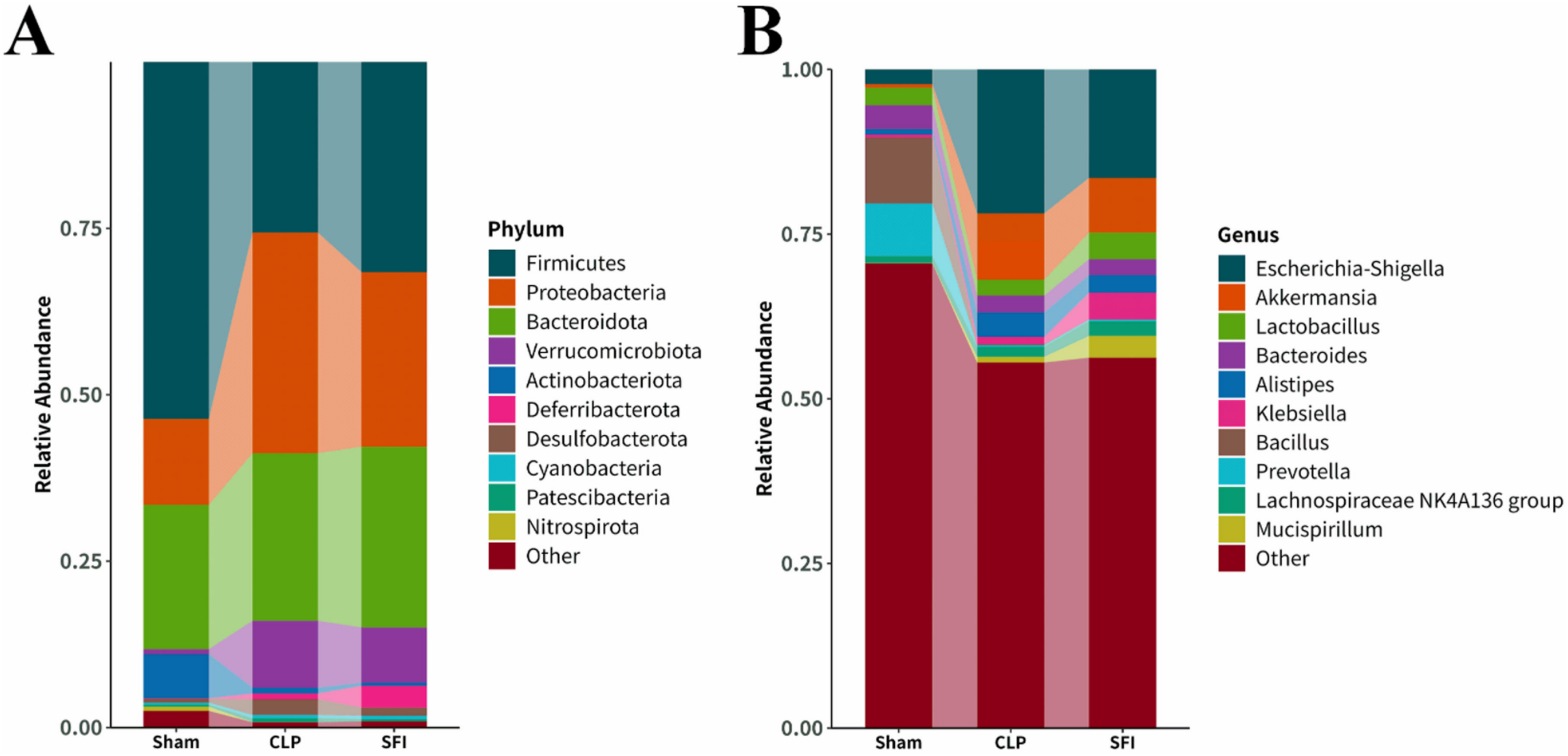
Composition of gut microbiota in mice 16 hours after surgery of CLP and SFI, as well as in the Sham group. **(A)** Phylum level. **(B)** Genus level.

To further investigate the effect of SFI treatment on sepsis, the differences in phylum and genus levels were analyzed. At the phylum level, Firmicutes, Proteobacteria, Bacteroidota, Verrucomicrobiota, and Actinobacteriota were predominant (together accounting for 95.6, 94.9, and 93.7% of the microbiota in the sham, CLP, and SFI, respectively). The relative abundance of dominant phyla changed 16 hours after CLP surgery. Compared with the sham group, the CLP group significantly increased the levels of Proteobacteria and Desulfobacterota and significantly decreased the levels of Firmicutes, Actinobacteriota, and Nitrospirota (*P*< 0.05). In the SFI-treated group, the most significant change was a significant increase in the level of Firmicutes and a significant decrease in the level of Proteobacteria compared with the CLP group (*P*< 0.05). In addition, the SFI-treated group significantly increased the levels of Verrucomicrobiota and Proteobacteria and significantly decreased the level of Actinobacteriota compared with the sham group (*P*< 0.05) (**Figure 4A**).

At the genus level, the dominant genera were classified as *Escherichia-Shigell*, *Akkermansia*, *Lactobacillus*, *Bacteroides*, *Alistipes*, *Klebsiella*, *Bacillus*, *Prevotella*, *Lachnospiraceae* NK4A136 group and *Mucispirillum* (together accounting for 29.45%, 44.50%, and 43.76% of the microbiota in the sham, CLP, and SFI groups, respectively). Sixteen hours after CLP surgery, the CLP group significantly increased the levels of *Klebsiella* and *Alistipes*, and significantly decreased the levels of *Bacillus*, *Lactobacillus*, *Bacteroides*, and *Prevotella* compared with the sham group (*P*< 0.05). In the SFI-treated groups, the SFI group significantly increased the levels of *Lactobacillus*, *Mucispirillum*, and *Lachnospiraceae* NK4A136 group, and significantly decreased the level of *Escherichia-Shigella* compared with the CLP group (*P*< 0.05). In addition, the abundance of *Akkermansia* and *Lactobacillus* was significantly increased, while the abundance of *Bacteroides* and *Bacillus* was significantly decreased in the SFI group compared with the sham group (*P*< 0.05) (**Figure 4B**).

**Figure 5:**
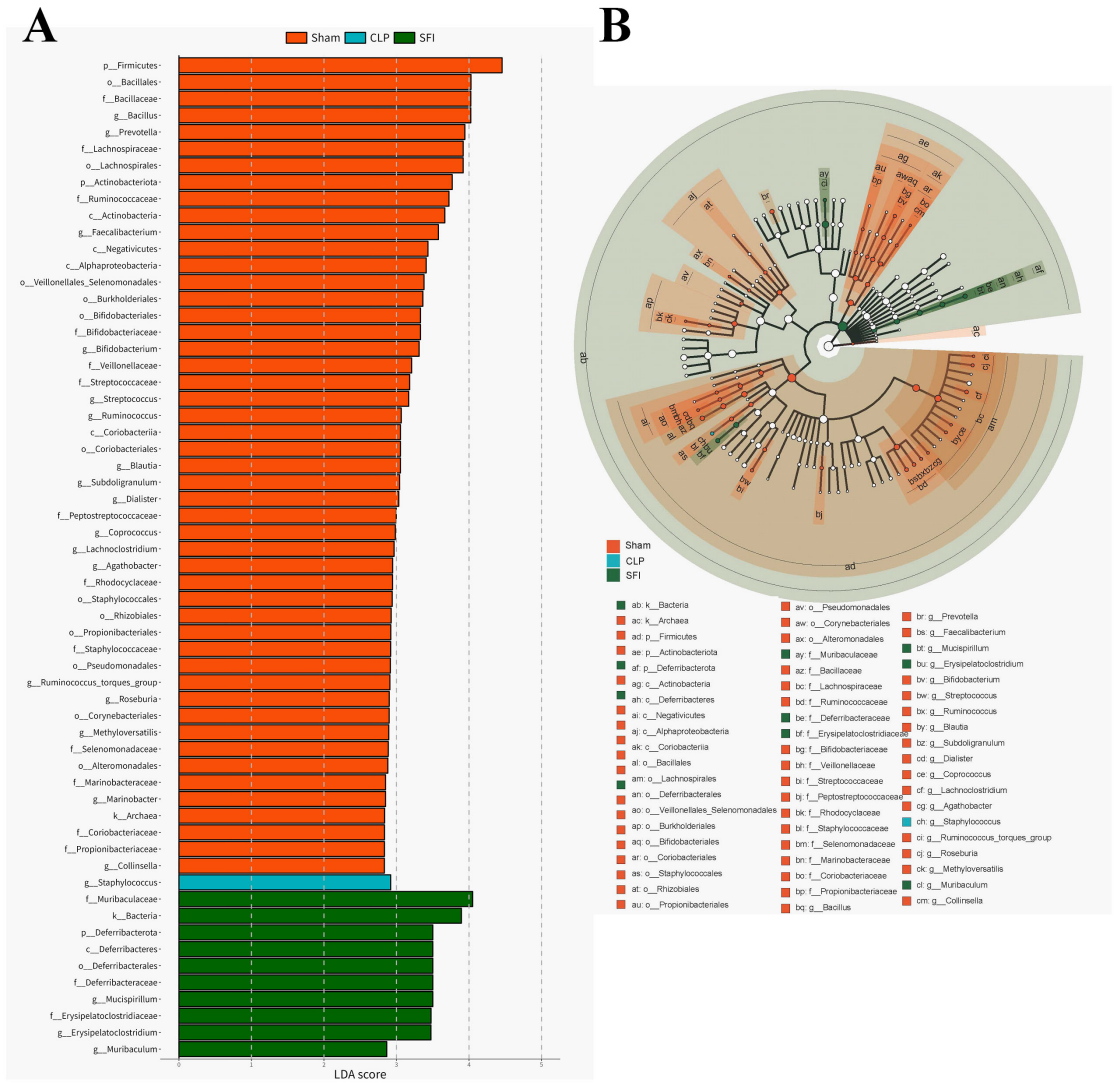
Characterization of gut microbiota in mice 16 hours after surgery of CLP and SFI, as well as in the Sham by LDA and LEfSe analysis. **(A)** Histogram of the LDA scores (log10) calculated for features differentially abundant in control, GOS, and RS samples (with LDA scores> 2.0). **(B)** Bacterial taxa differentially represented among groups identified by LEfSe.

Linear discriminant analysis (LDA) effect size (LEfSe) analysis (LDA score > 2.0) was performed to identify specific taxa that contributed to the differences between the three populations (**Figure 4**). A total of 60 bacterial taxa (49 in the sham group; 1 in the CLP group; and 10 in the SFI group) showed differences between the three populations (**Figure 5A**). Firmicutes and Bacillales were enriched in the control group, while Staphylococcus were enriched in the CLP group, and Muribaculaceae and Bacteria were enriched in the SFI group. Specifically, Muribaculaceae and Erysipelatoclostridium were enriched in the SFI, while Staphylococcus were enriched in the CLP. Cladogram analysis also revealed differences in bacterial taxa between the experimental groups (**Figure 5B**).

### 3.6 Associations between gut microbiota and inflammation

**Figure 6:**
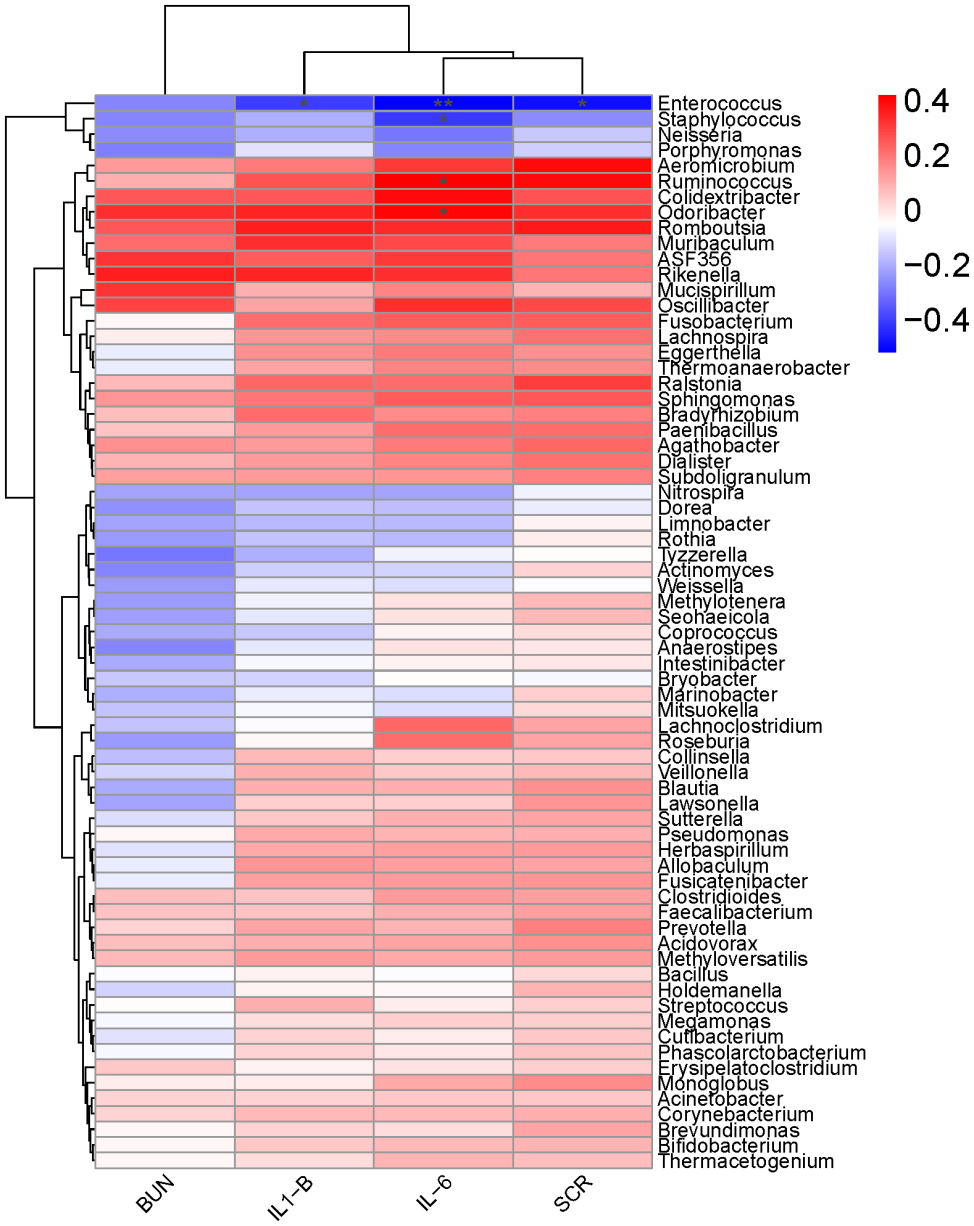
A heat map of Spearman’s correlation coefficients. Correlation between the abundance of key microbial taxa (at the genus levels) and the levels of inflammatory factors (IL-1β, IL-6, SCr and BUN) in mice 16 hours after surgery. Red and blue colors indicate positive and negative correlation coefficients, respectively. Significant correlations (P< 0.05) are indicated with an asterisk.

Spearman’s correlation analysis was used to determine the relationships between differentially abundant taxa (at the genus levels) and inflammation (**Figure 6**). The result showed that the expression level of IL - 1β was negatively correlated with the relative abundance of *Enterococcus* (Spearman’s ρ [rs] = -0.40, *P* = 0.04). IL - 6 had a significant positive correlation with the relative abundance of *Ruminococcus* (rs = 0.42, *P* = 0.04) and *Odoribacter* (rs = 0.40, *P* = 0.04), while negatively correlated with *Enterococcus* and *Staphylococcus*. Furthermore, a strong positive correlation was observed between SCR and *Enterococcus* (rs = -0.49, *P* = 0.01).

## 4 Discussion

In this study, a model of severe sepsis was established in mice using CLP (Rittirsch et al., 2009), and the effects of drug administration on intestinal epithelial cells and inflammation to assess whether SFI can restore the balance of the microbiota. Cecal ligation and puncture resulted in elevated heart rate, body temperature, and white blood cell, indicating systemic inflammation, which was not detected in the sham surgery. It also significantly increased serum levels of IL - 1β and IL - 6, liver and kidney function markers BUN and SCr levels. This model meets the criteria of the Diagnosis and Treatment Guidelines for Sepsis: 2012 (Dellinger et al., 2013), indicating that a severe sepsis mice model was successfully established.

Severe trauma or sepsis can induce accelerated apoptosis of intestinal mucosal epithelial cells, lamina propria lymphocytes, and eosinophils, thereby destroying the integrity of the intestinal barrier, leading to abnormally increased mucosal permeability and translocation of intestinal microbiota (Hotchkiss et al., 2016; Tao et al., 2024; Wang et al., 2024a; Yan et al., 2025). In the severe sepsis model that we established, the intestinal morphology changed significantly 16 hours after CLP surgery, with a large amount of necrotic cell fragments, necrotic and detached epithelial cells, and a small amount of capillary congestion observed in the intestinal cavity. These observations may be attributed to sepsis-induced systemic blood flow redistribution, which can lead to intestinal microcirculatory hypoperfusion, followed by hypoxic injury of intestinal mucosal epithelial cells (Duess et al., 2023). This pathological process further leads to increased mucosal endothelial permeability, enhanced leukocyte-endothelial cell adhesion, and degradation of tight junction proteins, ultimately resulting in dual dysfunction of the intestinal mechanical barrier and immune barrier (King et al., 2014).

In addition, in the early stage of sepsis, the NF-κB signaling cascade, triggers the positive feedback release of pro-inflammatory factors such as IL - 1β, IL - 6, and PAF, and upregulates the secondary infiltration of neutrophils mediated by IL - 8, forming an “inflammation-coagulation vicious cycle”, which ultimately leads to intestinal barrier collapse and multiple organ dysfunction (Kurt et al., 2007; Sun et al., 2021; Wang et al., 2021). As observed in our model, injury and bleeding may accelerate the inflammatory response of intestinal epithelial cells. At 8 h after CLP surgery, the levels of IL - 1β and IL - 6 in intestinal epithelial cells in the CLP group were significantly higher than those in the control group. SFI has previously been reported to alleviate inflammatory responses by inhibiting the NF-κB pathway (Liu et al., 2019; Wang et al., 2024b). In our model, SFI improved CLP-induced inflammatory responses by reducing IL - 1β and IL - 6 levels.

In patients with sepsis, serum creatinine (SCr) and blood urea nitrogen (BUN) are important indicators of renal function damage and are closely related to the severity and prognosis of the disease. Studies have shown that the BUN level of patients with sepsis is significantly higher than that of patients without sepsis (Gao et al., 2012; Min et al., 2022). Although there is no statistical difference in SCr between the two groups, it is positively correlated with oxidative stress factors such as malondialdehyde (MDA) and nitric oxide (NO), indicating that oxidative stress may aggravate renal function damage. The systemic inflammatory response and microcirculatory disorders caused by sepsis can lead to acute kidney injury (AKI), which in turn increases SCr and BUN, reflecting decreased glomerular filtration rate and azotemia (Costa et al., 2016; Qiu et al., 2019). In addition, the increase in BUN may also be related to a high metabolic state, increased protein breakdown, and insufficient renal perfusion. In our model, SCr and BUN levels in the blood were significantly increased by 5.5-fold and 4.4-fold at 16 h after CLP surgery. SFI significantly reduced SCr and BUN levels. These results suggest that SFI can prevent renal injury by regulating SCr and BUN levels, an observation previously reported in a rat model of intestinal ischemia-reperfusion (Hua et al., 2024) and heart failure (Zhao et al., 2020) after SFI into intestinal and cardiac tissues.

The intestine has long been considered a key factor in multiple organ dysfunction syndrome (MODS) (Klingensmith and Coopersmith, 2016; Mittal and Coopersmith, 2014; Van Coillie et al., 2022). Maintaining intestinal and systemic immune homeostasis depends on a balanced microbiota, and an imbalance in the intestinal microbiota may increase an individual’s risk of sepsis (Haak and Wiersinga, 2017). Regulating the intestinal flora may become a new direction for the treatment of sepsis (Haak et al., 2018). In critically ill patients, dysbiosis is common, manifested by a decrease in the number of “beneficial” commensal bacteria (such as Firmicutes or Bacteroidetes) and an enrichment of potentially pathogenic intestinal bacteria (such as Proteobacteria) (Sun et al., 2022; Wozniak et al., 2022). The abundance of Proteobacteria may become a potential indicator for disease diagnosis (Rizzatti et al., 2017; Shin et al., 2015). In addition, the intestinal microbiota is able to prevent foreign microorganisms from colonizing the gastrointestinal tract, a phenomenon known as “colonization resistance” (Kim et al., 2017). Studies have shown that *Escherichia coli*, *Proteobacterium*, and *Enterobacter* may cause bacteremia in frail patients because these intestinal bacteria are more prone to translocation, especially obligate anaerobes (Girard and Ely, 2007; Hyernard et al., 2019). Our study showed that there were significant differences in the intestinal microbial composition between the sham and CLP groups. As expected, Shenfu could regulate the abundance of *Muribaculaceae*, *Lachnospirales*, *Escherichia-Shigella*, and other bacteria, restoring them to levels similar to those in healthy mice. *Escherichia-Shigella* may cause sepsis-associated inflammation due to its ability to invade and damage the human colonic epithelium (Azimirad et al., 2020). *Lachnospirales* are butyrate-producing bacteria, anaerobes with probiotic properties, and play specific roles in metabolic diseases, inflammatory environments, and biotransformations (Xia et al., 2023; Yang et al., 2024). *Muribaculaceae* was significantly reduced in abundance in colitis mice and has an important role in microbiota homeostasis (Niu et al., 2022; Zhu et al., 2024). In addition, Interleukins can also interact with the microbiota in certain circumstances. For example, gut microbiota can influence the concentration of bile acid and the level of interleukin-22 to orchestrate polycystic ovary syndrome (Qi et al., 2019), *Akkermansia muciniphila* can improves cognitive function in aged mice by reducing the proinflammatory cytokine IL - 6 (Zhu et al., 2023). In this study, *Enterococcus* were significantly positive correlated with IL - 1β, IL - 6, SCR. A previous study has reported that antimicrobial overproduction sustains intestinal inflammation by inhibiting *Enterococcus* colonization (Jang et al., 2023), which demonstrate the relationship between *Enterococcus* and inflammation. In addition, the relative abundance changes of *Staphvlococcus, Ruminococcus, Odoribacten* were also significantly correlated with the expression of IL - 6, indicating that IL6 might be able to regulate the diversity changes of the bacterial community. As mentioned above, Shenfu can regulate the intestinal microbiota of septic mice by increasing beneficial bacteria and reducing pathogenic bacteria. However, although this article has to some extent expounded on the role of SFI in regulating the intestinal flora, the 16-hour observation window can only capture the acute-phase response and cannot reflect the long-term results. Therefore, in subsequent research and applications, if SFI is to be used clinically, further in-depth studies on its mechanism of action are still necessary.

## 5 Conclusions

In conclusion, this study established a cecal ligation and puncture (CLP) model to evaluate the therapeutic effects of Shenfu Injection (SFI) in sepsis management. The experimental results demonstrated that SFI administration significantly attenuated intestinal inflammation and reduced serum levels of pro-inflammatory mediators, including IL - 1β, IL - 6, SCr, and BUN. Furthermore, microbial analysis revealed that SFI treatment effectively modulated gut microbiota composition by decreasing the relative abundance of pathogenic bacteria (particularly *Escherichia-Shigella*) while enhancing probiotic populations, notably *Akkermansia* and *Lactobacillus* species. These findings collectively indicate the therapeutic potential of SFI in sepsis treatment through dual mechanisms of inflammatory response mitigation and gut microbiota regulation. Notably, this investigation provides a valuable foundation for future mechanistic studies exploring SFI-mediated sepsis management via intestinal microbiome modulation, potentially informing the development of novel therapeutic strategies for critical care medicine.

## Data Availability

The datasets used and/or analyzed during the current study are available from the corresponding author on reasonable request. Requests to access these datasets should be directed to a8lg11@163.com.
